# Zero-Dimensional Cs_3_BiX_6_ (X = Br, Cl) Single Crystal Films with Second Harmonic Generation

**DOI:** 10.1186/s11671-022-03759-4

**Published:** 2022-12-07

**Authors:** Junjie Yao, Zhicheng Zhou, Lutao Li, Yuan Chen, Chen Wang, Xiangyi Wang, Zheng Lu, Zhongchao Bai, Qiang Zhang, Xuefeng Huangfu, Yinghui Sun, Hao Xu, Guifu Zou

**Affiliations:** 1 School of Energy, Key Laboratory of Advanced Carbon Materials and Wearable Energy Technologies of Jiangsu Province, Soochow University, Suzhou, 215006 People’s Republic of China; 2 College of Mechanical and Electronic Engineering, Shandong University of Science and Technology, Qingdao, 266590 People’s Republic of China; 3 School of Physics, University of Electronic Science and Technology of China, Chengdu, 610054 People’s Republic of China; 4 Yangtze Delta Region Institute (Huzhou), University of Electronic Science and Technology of China, Huzhou, 313001 People’s Republic of China

**Keywords:** Zero-dimensional, Cs_3_BiBr_6_, Cs_3_BiCl_6_, Single crystal film, Second harmonic generation

## Abstract

The development of atomically thin single crystal films is necessary to potential applications in the 2D semiconductor field, and it is significant to explore new physical properties in low-dimensional semiconductors. Since, zero-dimensional (0D) materials without natural layering are connected by strong chemical bonds, it is challengeable to break symmetry and grow 0D Cs_3_BiX_6_ (X = Br, Cl) single crystal thin films. Here, we report the successful growth of 0D Cs_3_BiX_6_ (X = Br, Cl) single crystal films using a solvent evaporation crystallization strategy. Their phases and structures are both well evaluated to confirm 0D Cs_3_BiX_6_ (X = Br, Cl) single crystal films. Remarkably, the chemical potential dependent morphology evolution phenomenon is observed. It gives rise to morphology changes of Cs_3_BiBr_6_ films from rhombus to hexagon as BiBr_3_ concentration increased. Additionally, the robust second harmonic generation signal is detected in the Cs_3_BiBr_6_ single crystal film, demonstrating the broken symmetry originated from decreased dimension or shape change.

## Introduction

Zero-dimensional (0D) inorganic perovskites offer a unique opportunity to investigate and decipher the charge carrier behavior in an intrinsically strong quantum-confined environment [1]. These reduced-dimensional perovskites have attracted a growing number of experimental and theoretical research groups around the world because of their high thermal and chemical stability as compared to their 3D analogues [2]. Atomically thin single crystal films have shown prospective applications in two-dimensional (2D) semiconductor device fields, such as field effect transistors, [3] photodetectors [4–7], phototransistors [8], light emitting diodes, [9, 10] laser diodes, [11] resistive random access memories, [12, 13] etc. Some novel physical properties can be observed upon the reduction of crystalline dimensions. For instance, Ciarrocchi et al. [14] reported the transition from metal to semiconductor by modulating thickness of transition metal dichalcogenides. Zhang et al. [15] revealed the transition from direct band gap to indirect band gap in atomically thin Arsenene and Antimonene. Wang et al. [16] observed the increased superconducting Curie temperature in high-quality monolayer superconductor NbSe_2_ grown by chemical vapor deposition. Cheema et al. [17] addressed the enhanced ferroelectricity in ultrathin-doped hafnium oxide grown directly on silicon. Huang et al. [18] studies the layer-dependent ferromagnetism in a van der Waals crystal down to the monolayer limit. Zhang et al. [19] have the experimental observation of the quantum Hall effect and Berry’s phase in graphene. Therefore, it is of great significance to develop new atomic layer semiconductor single crystal films [20–24].

In recent years, halide perovskites have drawn attentions due to their excellent light absorption coefficient, low trap state density, high carrier mobility, long carrier diffusion distance, as well as high defect tolerance factor [25–28]. It has been widely recognized as one of the most promising materials in future optoelectronic field. Inspired by the tremendous progress of 2D semiconductors, researchers spare much effort to explore their dimension dependent properties, [29–35] especially when its thickness is reduced to atomically thin [3, 26, 36, 37]. Currently, the atomically thin single crystal films of lead halide perovskites have been intensively investigated, including cubic, orthorhombic, and Ruddlesden–Popper structures [38–41]. As well known, the lead-free perovskites are one of the most important branches in the perovskites family due to environmental friendly and higher stability as well as excellent optoelectronic properties. [29, 30, 42, 43] Particularly for the Bi-based perovskites derivatives, [44–46] Cs_3_Bi_2_X_9_ has been demonstrated the potential applications for solar cells, light emitting diodes, photodetectors, etc. Although the Cs_3_BiX_6_ bulk and quantum dots have been synthesized and employed for x-ray scintillation and photodetectors, [47–50], there is still few report focused on the growth of Cs_3_BiX_6_ (X = Br, Cl) single crystal films.

Here, we report the growth of zero-dimensional Cs_3_BiX_6_ (X = Br, Cl) single crystal films using a solvent evaporation crystallization strategy. Their phase and structures are well evaluated to confirm zero-dimensional Cs_3_BiX_6_ (X = Br, Cl) single crystal films. It is revealed that the chemical potential dependent morphology evolution phenomenon makes the morphology of Cs_3_BiBr_6_ film change from rhombus to hexagon, as the BiBr_3_ concentration increased. A robust second harmonic generation signal was observed in the Cs_3_BiBr_6_ single crystal film, which demonstrated the broken symmetry due to decreased dimension or shape change. This work not only provides a single crystal film growth method for zero-dimensional structural materials, but also conducive to understanding the electron and band structure change as thickness reduced. This cross-disciplinary research represents a remarkable advance in nanomaterials science, chemistry and crystal growth.

## Experiment Details

### ***Grown of Atomically Thin Cs***_***3***_***BiBr***_***6***_*** and Cs***_***3***_***BiCl***_***6***_*** Single Crystal Films***

The thin Cs_3_BiBr_6_ single crystal film is synthesized from a drop casting and solvent evaporation crystallization process. The substrate for the films growing is Si/SiO_2_, briefly, 15 mM CsBr and 10 mM BiBr_3_ are firstly dissolved in 1 mL dimethyl sulfoxide, then, 0.1 mL *n*-octanoic acid is added and mixing uniformly. Subsequently, 20 μL above transparent solution is dropped onto cleaned SiO_2_/Si substrate and heated to 80 °C for 30 min, as the solution reach supersaturation, the nucleation and growth occurs. Finally, the films are produced on the substrate. For the Cs_3_BiCl_6_ single crystal film growth, all of the procedures are kept the same as Cs_3_BiBr_6_ film, only replace the CsBr and BiBr_3_ with CsCl and BiCl_3_, respectively.

### Characterization

Optical microscopy images are taken using a Nikon ECLIPSE LV150N microscopy; SEM images are taken using a Hitachi (SU-8010) which equipped with a x-ray energy dispersion spectra; TEM images are recorded from (F20, 200 kV); X-ray diffraction pattern is taken in x-ray diffractometer (Bruker D8 Advance); XPS patterns are taken in x-ray photoelectron spectroscopy (THERMO FISHER, ESCALAB 250Xi); AFM images were taken by a Bruker Multimode8 AFM system; The Raman spectra are measured by Raman spectrometer (Horiba Jobin Yvon HR Evolution, the excitation light source is a 532 nm laser). The UV–vis optical spectra are measured by ultraviolet visible spectrophotometer (UV 2450, Shimadzu).

### SHG Measurement

The SHG laser pulse duration was 10 s, and the laser spot was 1 μm × 1 μm. The fundamental frequency signal is focused onto the sample by using an pulsed laser. A long-pass dichroic mirror separates the reflected fundamental signal from the second harmonic signal. The SHG is directed onto a tube lens and projected onto a CCD camera, forming the image of the SHG signal.

## Results and Discussion

**Fig. 1:**
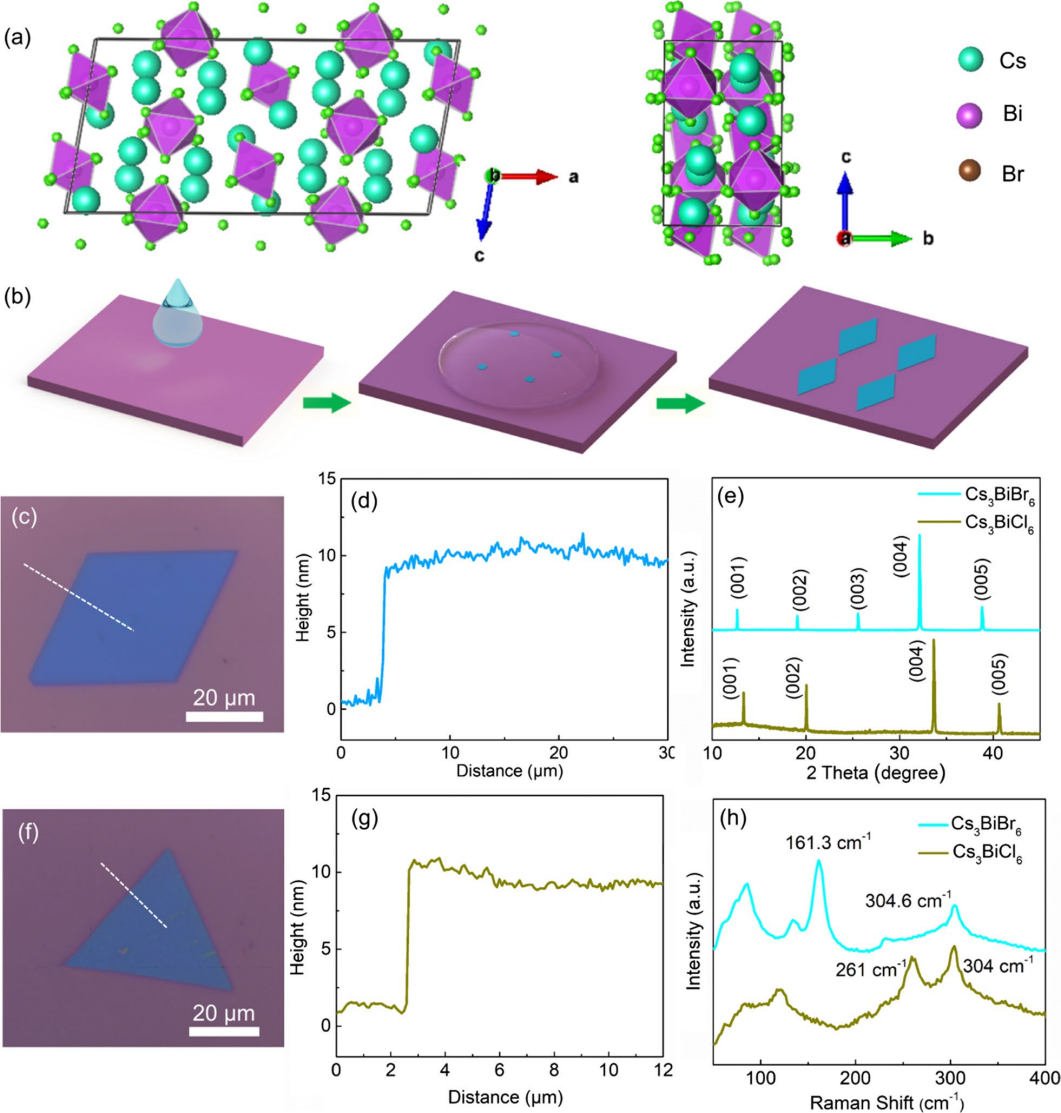
**a** Schematic diagram of the Cs_3_BiBr_6_ zero-dimensional structure: top view (left) and side view (right). **b** Schematic procedure of Cs_3_BiBr_6_ film growth. **c** Optical microscope image of the obtained Cs_3_BiBr_6_ films. **d** Height profile obtained from the AFM profile image of the Cs_3_BiBr_6_ film in (**c**). **e** XRD patterns of the Cs_3_BiBr_6_ and Cs_3_BiCl_6_ films. **f** Optical microscope image of the obtained Cs_3_BiCl_6_ films. **g** Height profile obtained from the AFM profile image of the Cs_3_BiBr_6_ film in (**f**). **h** Raman spectra of the Cs_3_BiBr_6_ and Cs_3_BiCl_6_ films

As studied, the Cs_3_BiBr_6_ presents a monoclinic phase at room temperature, and the corresponding space group is C2/c, whose lattice parameters are: *a* = 28.31 Å, *b* = 8.617 Å, *c* = 13.71 Å, *β* = 99.48°, unit cell volume = 329.8 Å^3 (Fig. 1a left) [47]. Notably, the [BiBr_6_]^3−^ regular octahedrons are isolated from each other and disconnected from the vertex bromine atoms (Fig. 1a right), resulting in electrons and holes localized in the [BiBr_6_]^3−^ octahedrons. Therefore, it is usually defined as the zero-dimensional structure. Herein, a solvent evaporation crystallization strategy is adopted to grow the thin films. Briefly, CsBr and BiBr_3_ are dissolved in dimethyl sulfoxide. Then, a drop of precursor solution is dropped onto clean substrate accompanying with being heated to evaporate the solvent. As the solution reaches supersaturation, the nucleation and growth process were occurred. Finally, the well-defined thin films can be synthesized on the substrate (the synthesis process is illustrated in Fig. 1b, and the details are described in experimental section).

The morphology of the film is investigated by optical microscope. As shown in Fig. 1c, regular rhombus morphology with four long sides is observed on the Si/SiO_2_ substrate. The film shows blue color, while the thickness of film is estimated to be around 9 nm by atomic force microscopy (Fig. 1d). As the precursor metal halide salts changed from -Br to -Cl, the counterparts Cs_3_BiCl_6_ films with thickness of about 9 nm exhibit the morphology varying from regular rhombus to regular triangle and hexagon (Fig. 1f and g). To identify their crystal structures, the x-ray diffraction (XRD) patterns were obtained, as shown in Fig. 1e. The Br component related sample shows five clear and sharp peaks located at 12.69°, 19.12°, 25.56°, 32.13°, and 38.84°, corresponding to the (400), (600), (800), (1000), and (1200) facets, respectively. Reasonably, the periodic diffraction peaks can indicate good orientation of Cs_3_BiBr_6_. According to the Bragg diffraction formula: 2*d*·sin*θ* = *nλ*, where the *θ* is half of the diffraction peaks position (*θ* = 6.345°), *λ* is the wavelength of the Cu Kα (1.5406 Å), the lattice spacing is calculated to be ~ 6.97 Å. This parameter agrees well with the theoretical value of 7.07 Å. Similarly, the sample containing Cl component presents the clear peaks located at 13.367°, 20.047°, 33.655°, 40.655°, and 47.365°, corresponding to the (400), (600), (1000), (1200), and (1400) facets of Cs_3_BiCl_6_, respectively. Compared with the Cs_3_BiBr_6_ sample, all the peaks of Cs_3_BiCl_6_ are moved toward higher diffraction angles. It is result from the smaller atomic radius of Cl that induced smaller lattice parameters and spacing. Moreover, the lattice spacing can be calculated to be ~ 6.695 Å by the Bragg diffraction formula. Furthermore, the Raman spectroscopy was employed to investigate phonon oscillation of the film. As shown in Fig. 1g, two clear vibration peaks of Cs_3_BiBr_6_ resolved at 304.6 cm^−1^ and 161.3 cm^−1^ are attributed to [BiBr_6_]^3−^ complexes. Since the [BiBr_6_]^3−^ is not corner shared, the vibration peaks present asymmetric broadening. At the same time, the Raman spectrum of Cs_3_BiCl_6_ show the two broad peaks around 261 cm^−1^ and 304 cm^−1^ (Fig. 1g), similar to the Cs_3_BiBr_6_ sample.

**Fig. 2:**
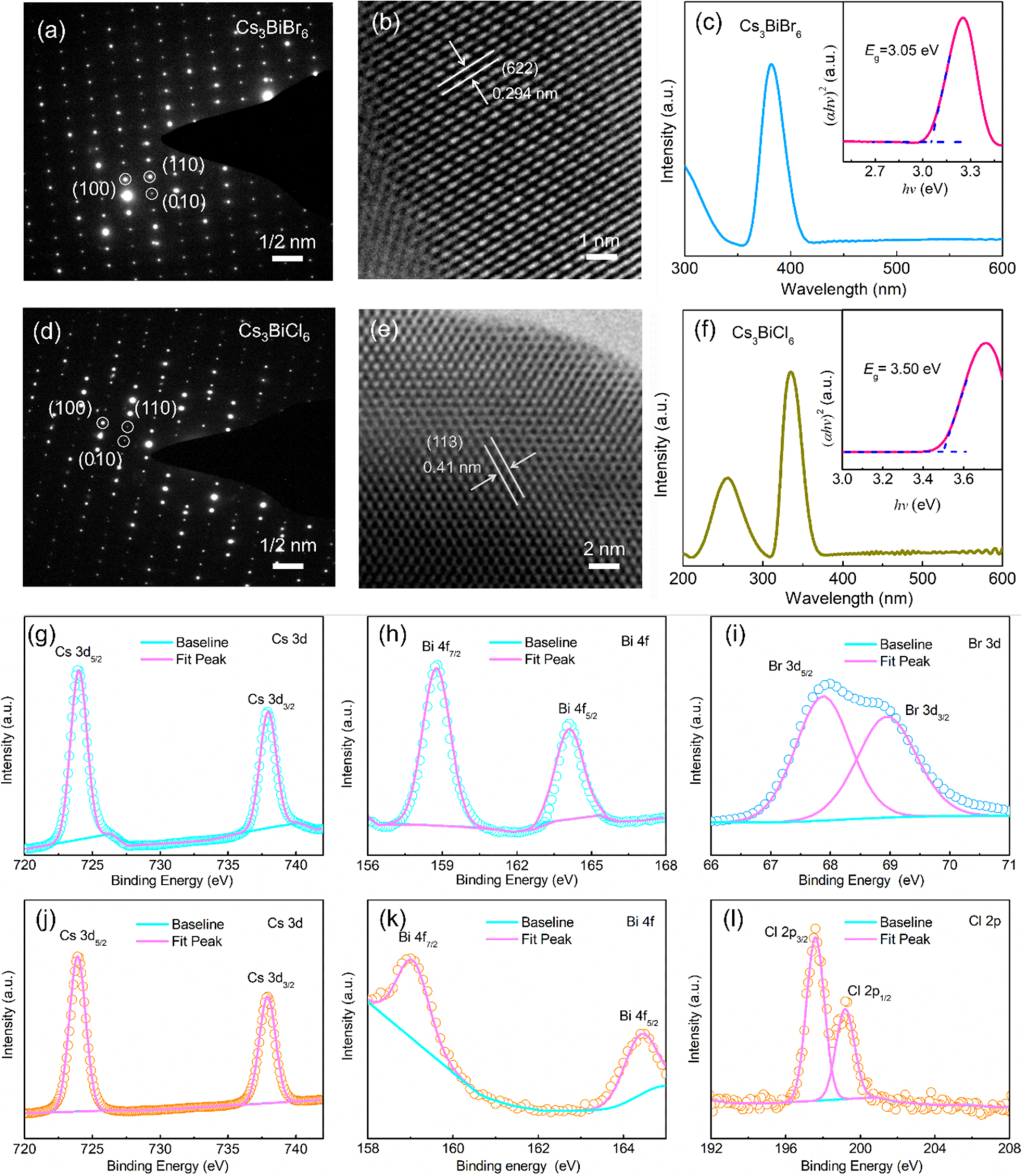
**a** Selected area electron diffraction pattern of the Cs_3_BiBr_6_ film. **b** HRTEM image of the Cs_3_BiBr_6_ film. **c** UV–vis absorption spectra of the Cs_3_BiBr_6_ film, the insert is the band gap value converted with the Tauc Plot method. **d** Selected area electron diffraction pattern of the Cs_3_BiCl_6_ film. **e** HRTEM image of the Cs_3_BiCl_6_ film. **f** UV–vis absorption spectra of the Cs_3_BiCl_6_ film, the insert is the band gap value converted with the Tauc Plot method. XPS high-resolution scanning spectra of the of the Cs_3_BiBr_6_ film: **g** Cs 3d, **h** Bi 4f, **i** Br 3d. XPS high-resolution scanning spectra of the of the Cs_3_BiCl_6_ film: **j** Cs 3d, **k** Bi 4f, **l** Cl 2p

To further investigate the microstructure and crystallization of the film, Fig. 2a shows the Selected Area Electron Diffraction (SAED) pattern of the Cs_3_BiBr_6_ sample. The clear lattice image without diffractive ring was observed, suggesting the single crystalline features in the local area. The diffraction spots can be labelled (100), (110), and (010) facets, respectively. Figure 2b presents the high-resolution TEM (HRTEM) with clear and long range order lattice It is measured to be ~ 0.294 nm, corresponding to the (622) facet of the Cs_3_BiBr_6_. Due to the different atomic radius, the SAED pattern of Cs_3_BiCl_6_ presents more obvious zero-dimensional structure features whose diffraction intensity periodically changes along the same crystal orientation axis (Fig. 2d). Simultaneously, the HRTEM of Cs_3_BiCl_6_ in Fig. 2e shows the lattice fringe spacing of ~ 0.41 nm, corresponding to the (113) facet. Furthermore, the optical absorption is a very important feature for Cs_3_BiX_6_ single crystal films. In terms of Cs_3_BiBr_6_, Fig. 2c shows a sharp cutoff edge at around 415 nm and a strong absorption peak at around 381 nm. Reasonably, the excitation adsorption feature of the Cs_3_BiBr_6_ addresses a direct bandgap semiconductor with the bandgap value of ~ 3.05 eV via the Tauc plot method (Fig. 2c insert). As for the Cs_3_BiCl_6_ single crystal film, it shows a sharp cutoff edge at around 370 nm and a strong excitation adsorption peak central at around 335 nm in Fig. 2f, indicating a direct bandgap semiconductor feature with the bandgap value of ~ 3.5 eV via the Tauc Plot method (Fig. 2f insert). The calculated bandgap of Cs_3_BiX_6_ films agree well with the previous Cs_3_BiX_6_ quantum dots in the literature [47]. To investigate the element valance state, Fig. 2g–i shows the XPS high-resolution scanning spectra of the Cs_3_BiBr_6_ single crystal film. The binding energy located at 158.8 eV and 164.1 eV for Bi 4f, 723.96 eV and 737.95 eV for Cs 3d, 67.89 eV and 68.96 eV for Br 2p can be assigned to be the Cs^+^, Bi^3+^, and Cl^−^, respectively. Meanwhile, Fig. 2j–l shows the binding energy located at 159 eV and 164.4 eV for Bi 4f, 724.3 eV and 738.2 eV for Cs 3d, 197.5 eV and 199.2 eV for Cl 2p, corresponding to the Cs^+^, Bi^3+^, and Cl^−^ of the Cs_3_BiCl_6_ single crystal film, respectively. Compared with the Br component sample, the XPS high-resolution spectra of Bi 4f and Cs 3d in Cl component sample have more obvious shift toward higher binding energy. It can be due to the shorter bond length and stronger bond energy than the Br component sample, and the change in valence state is consistent with the slight difference in their intrinsic structure.

**Fig. 3:**
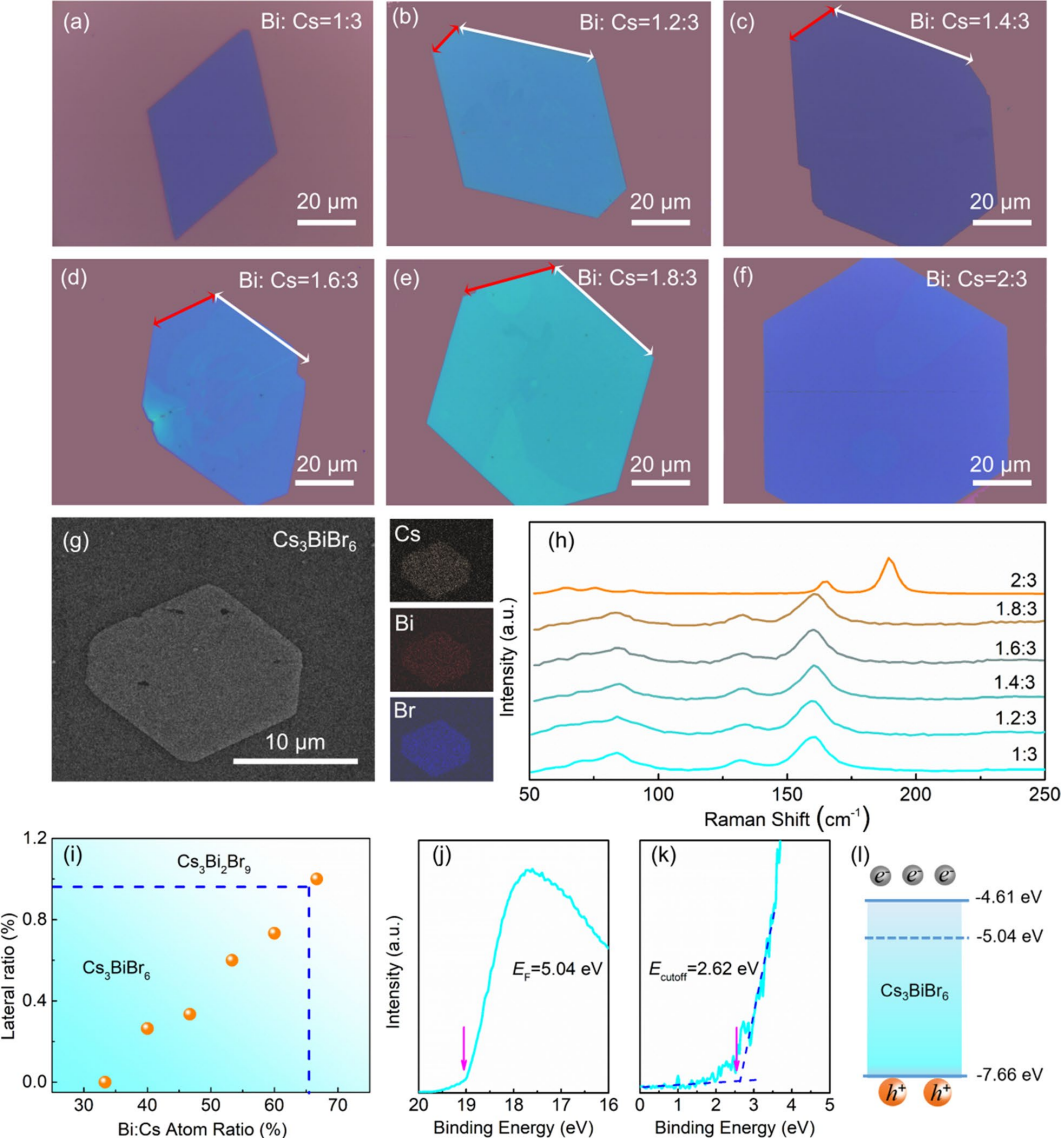
Optical microscope images of the Cs_3_BiBr_6_ film grown with varied Bi: Cs ratio: **a** 1: 3, **b** 1.2: 3, **c** 1.4: 3, **d** 1.6: 3, **e** 1.8: 3, **f** 2: 3, respectively. **g** EDS mapping of the Cs_3_BiBr_6_ film grown with the Bi:Cs ratio of 1.6:3. **h** Raman spectra of the Cs_3_BiBr_6_ film grown with varied Bi:Cs ratios. **i** Statistical image of film morphology changing with Bi:Cs ratios, the different colors in the figure represents Cs_3_BiBr_6_ and Cs_3_Bi_2_Br_9_ phases region, respectively. **j**, **k** UPS patterns of the Cs_3_BiBr_6_ film. **l** Schematic band structure diagram of the Cs_3_BiBr_6_ film

Apart from the Cs_3_BiBr_6_ components, it is noticed that the cesium bismuth halide compounds have another phase Cs_3_Bi_2_Br_9_ with higher BiBr_3_ stoichiometric ratio and another crystal structure. To further study the evolution and control mechanism of the two-phase, more systematic experiments were carried out, including varying proportions of Bi: Cs in the precursor. As the percent of Bi:Cs is changed from 33 to 60% in Fig. 3a–f, the shape of the film shows obvious transformation from the initial rhombus with four sides to the following hexagon along with the two edge length increased. As well known, the concentration has a huge influence on chemical potential, thus it could suggest that the chemical potential of Bi plays a significant role during the film morphology transformation from rhombus toward hexagon. In addition, Fig. 3g illustrates energy dispersion spectroscopy mapping results of the samples. Cs, Bi and Br elements in the samples are distinguished and distributed uniformly, which could be estimated to be the elements proportion of 2.6: 1: 7.1. The ratio is close to the Cs_3_BiBr_6_ phase. The Raman spectroscopy measurement is used to further evaluate the composition. In Fig. 3h, when the ratio is below 2:3, the peak intensity and wavenumber of the peaks resolved around 160 cm^−1^ and 132 cm^−1^ did not show evident difference among the samples. This indicates that the morphological variation should be not accompanied with structure phase transition. Furthermore, as the Bi:Cs further rise to 2:3 (67%), the Cs_3_Bi_2_Br_9_ phase can dominate crystallization process, while two-phase coexistence phenomenon cannot be found based on the Raman spectra. Reasonably, it should be due to the higher forming energy barrier of Cs_3_Bi_2_Br_9_ phase. Consequently, the phase and lateral size as a function of Bi proportion is extracted and statistics in Fig. 3i, it exhibiting obvious chemical potential dependency on BiBr_3_. Furthermore, the detailed band structure of the Cs_3_BiBr_6_ is investigated from ultraviolet photoelectron spectroscopy (Fig. 3j and k), and the Fermi level and *E*_cutoff_ are − 5.04 eV and 2.62 eV, respectively. The valance band maximum and conduct band minimum are estimated to be − 7.66 eV and − 4.61 eV, respectively (as illustrated in Fig. 3l). As shown, the Fermi level is close to conduct band bottom, suggesting n-type semiconductor features.

**Fig. 4:**
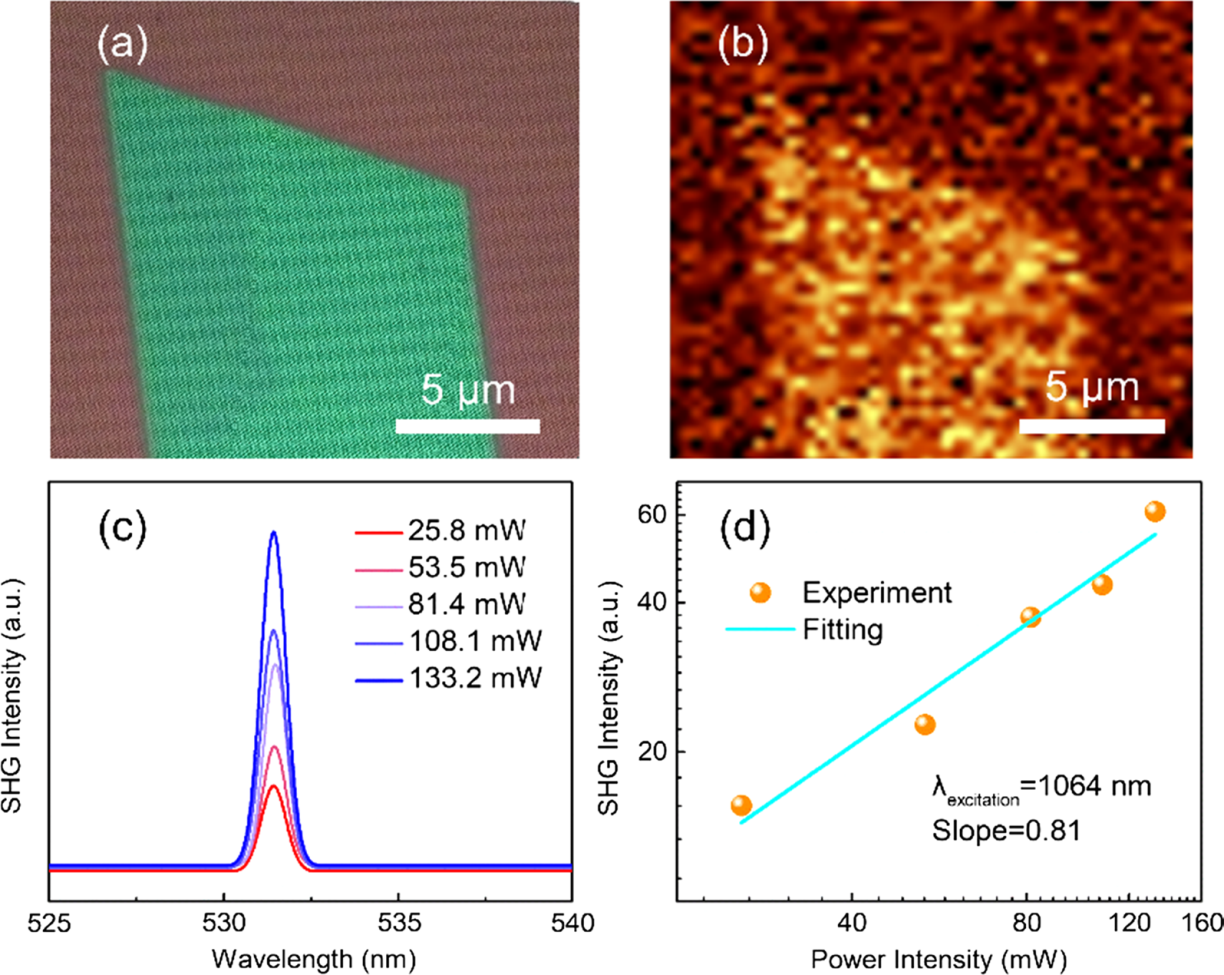
**a** Optical microscope image of the Cs_3_BiBr_6_ film that used for SHG measurement. **b** SHG mapping image of the rhombus sample in (**a**). **c** Second harmonic generation properties of the Cs_3_BiBr_6_ film with varied excitation power. **d** Statistical image of SHG intensity as a function of excitation power intensities

Considering the crystal structure of Cs_3_BiBr_6_ exhibiting spatial symmetry, lattice parameters (such as bond length and bond angle) could change as the thickness decreased down to atomic level and morphology transition. To further identify the above cases, the second harmonic generation (SHG) measurements for the Cs_3_BiBr_6_ film were performed. A 1064 nm laser is used as excitation light source. Firstly, no SHG signal is observed in the thick film (about 640 nm) of the rhombus sample, while the region with smaller thickness film (about 39 nm) shows SHG signals (Fig. 4a and b). In addition, for the Cs_3_BiBr_6_ hexagonal film, as shown in Fig. 4c, there is a doubling frequency signal at 532 nm and the intensity is regularly increased as the laser power increasing from 25.8 mW to 133.2 mW. Figure 4d shows the statistics result of SHG intensity as a function of laser power, illustrating the linear relationship with a slop of 0.81 by a parabolic relationship (*I* ∝ *P*^*θ*^). According to the electric dipole approximation theory [51], the second harmonic signal can be only generated in the medium with asymmetric center. Therefore, it indicates that there is an obvious lattice parameter variation, forming an asymmetric structure in the film. To sum up, as the dimensionality of Cs_3_BiBr_6_ is decreased or shape changed to hexagonal), symmetry can tend to be easily broken. This work might provide a thought about the growth of low-dimensional materials and show further influence on electron/band structures.

## Conclusion

In conclusion, we report the successful growth of zero-dimensional Cs_3_BiX_6_ (X = Br, Cl) single crystal films with a solvent evaporation crystallization strategy. It was found that solution concentration is strongly correlated with chemical potential, which plays a key role on morphology evolution from rhombus to hexagon. A robust second harmonic generation signal demonstrates the broken symmetry, attributed to decreased dimension or shape change. This work not only provides a single crystal film growth method for zero-dimensional structural materials, but also conducive to understanding the electron and band structure change as its thickness reduced.

## Data Availability

All datasets are presented in the main paper and freely available to any scientist wishing to use them for non-commercial purposes, without breaching participant confidentiality.
